# Strain Engineering of Correlated Charge-Ordered Phases in 1T-TaS_2_

**DOI:** 10.1021/acs.nanolett.5c04101

**Published:** 2025-10-30

**Authors:** Rafael Luque Merino, Felix Carrascoso, Eudomar Henríquez-Guerra, M. Reyes Calvo, Riccardo Frisenda, Andres Castellanos-Gomez

**Affiliations:** † 2D Foundry Research Group, 69570 Instituto de Ciencia de Materiales de Madrid (ICMM-CSIC), Madrid E28049, Spain; ‡ BCMaterials, Basque Center for Materials, Applications and Nanostructures, 48940 Leioa, Spain; § IKERBASQUE, Basque Foundation for Science, 48009 Bilbao, Spain; ∥ Dipartimento di Fisica, Università di Roma “La Sapienza”, I-00185 Roma, Italy

**Keywords:** strain engineering, charge density wave, piezoresistance, phase transition, strain sensor

## Abstract

Strain engineering is a powerful strategy for controlling the structural and electronic properties of two-dimensional materials, particularly in systems hosting charge density wave (CDW) order. In this work, we apply uniaxial tensile and compressive strain to thin 1T-TaS_2_ flakes using a flexible, device-compatible platform and systematically investigate the strain-dependent behavior of the nearly commensurate (NC) to incommensurate (IC) CDW phase transition. This transition is driven by Joule heating at room temperature. Electrical transport measurements reveal that both the switching threshold voltage and the resistance of the NC-CDW phase exhibit clear, reversible strain dependence. Furthermore, we identify a quadratic dependence between the strain-induced resistance change and the threshold voltage, confirming that piezoresistive modulation governs the strain tunability of the phase transition. We demonstrate a room-temperature, electrical readout strain and displacement sensor with a threshold-like response in a programmable window. These results highlight the potential of 1T-TaS_2_ for on-chip sensing of strain and displacement.

## Graphical abstract

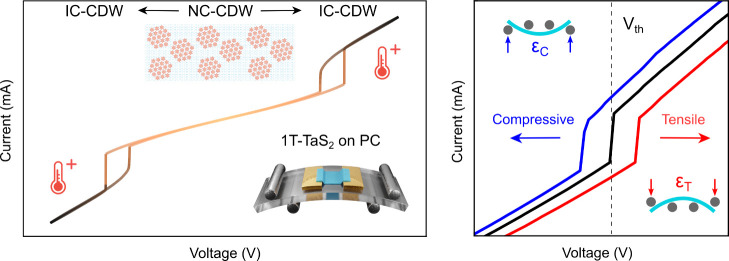

Charge density waves (CDW) are periodic modulations of the electronic charge density, coupled to lattice distortions.^1^ CDWs generally emerge due to electron–electron interactions (via Fermi surface nesting)^1,2^ and electron–phonon interactions (with an associated CDW phonon mode).^3,4^ Materials hosting CDW order are of great interest due to their rich phase diagrams, where the charge order is intimately linked to correlated, low-temperature phases like superconductivity^5,6^ and Mott physics.^7–9^ Two-dimensional (2D) materials hosting CDWs are particularly appealing, as charge order can persist above room temperature^10^ (RT) and the reduced dimensionality boosts their susceptibility to external perturbations.^11^

Strain engineering in 2D materials provides a versatile platform to modulate material properties: from bandgap engineering leading to changes of electronic and optical properties;^12^ to tipping the balance between ground states in correlated 2D materials.^13–16^ Within correlated materials, strain constitutes a natural tuning knob for CDW materials, as the charge order itself is coupled to a lattice distortion and thus highly sensitive to mechanical deformation. Strain engineering of CDW materials has been shown to alter the ground state,^7,17^ enhance or suppress charge order^18–22^ or modify the dynamics of metastable states.^23–25^ However, most studies rely on bulky hardware incompatible with integrated devices.^26–29^ Wrinkling via patterned substrates offers an on-chip alternative, yet it imposes only fixed, nontunable strain,^30,31^ similar to unintentional strain resulting from growth processes.^32^ These limitations highlight the opportunity to harness large, well-defined strain in device-compatible geometries to manipulate CDW order *in situ* and unlock novel electronic functionalities.

1T-TaS_2_, a layered transition metal dichalcogenide, is an attractive platform for tailoring CDW order because it hosts a hierarchy of CDW phases: from incommensurate (IC) order at high temperatures, to nearly commensurate (NC), to fully commensurate (C) order at low temperatures. Extensive work demonstrates that the individual CDW phases, and the transitions linking them, can be actively manipulated through a multitude of external perturbations, such as electrostatic gating,^33,34^ electromagnetic fields,^35–43^ targeted chemical doping^44–46^ and others.^47,48^ Of particular relevance for applications, the NC-to-IC phase transition occurs around T_C_ ≈ 350 K, opening the door to practical devices that exploit CDW phase transitions near RT.^35,36,40,41,49,50^

Previous studies of strain in 1T-TaS_2_, many of which focused on hydrostatic pressure,^28,51^ revealed dramatic effects such as a collapse of the Mott gap,^52,53^ control over metastable CDW phases,^31,54^ or the emergence of superconductivity.^28,51^ A handful of experiments have also explored in-plane deformations, but they rely on complex and resource-intensive setups^27–29^ or fixed-geometry approaches^30,31^ that preclude dynamic tuning. Crucially, most reports target the low-temperature phases, leaving the NC and IC orders near 350 K largely unexamined under strain. Dynamical strain-tuning of this phase transition could enable room-temperature, device-compatible CDW functionalities.

In this work, we employ a simple yet versatile method to apply uniaxial strain continuously and reversibly, enabling dynamic control of the NC-to-IC CDW transition in 1T-TaS_2_. This approach not only provides a practical route for exploring strain-dependent behavior but also lays the foundation for the development of strain-sensitive devices. To illustrate this potential, we demonstrate room-temperature electrical detection of both tensile and compressive strain in two distinct operation modes. Remarkably, one can leverage the CDW phase transition as an intrinsic amplification mechanism to obtain exceptionally large strain sensitivity.

Figure 1a illustrates the experimental concept, where uniaxial strain is applied to a 1T-TaS_2_ device fabricated on a flexible polycarbonate substrate using a four-point bending setup.^55^ A mechanically exfoliated, thin flake is positioned onto prepatterned source and drain electrodes, forming the conductive channel (Figure 1b). The four-point bending configuration allows for controlled application of tensile (*ε*_*T*_) and compressive (*ε*_*C*_) strain directly during electrical measurements.

**Figure 1:**
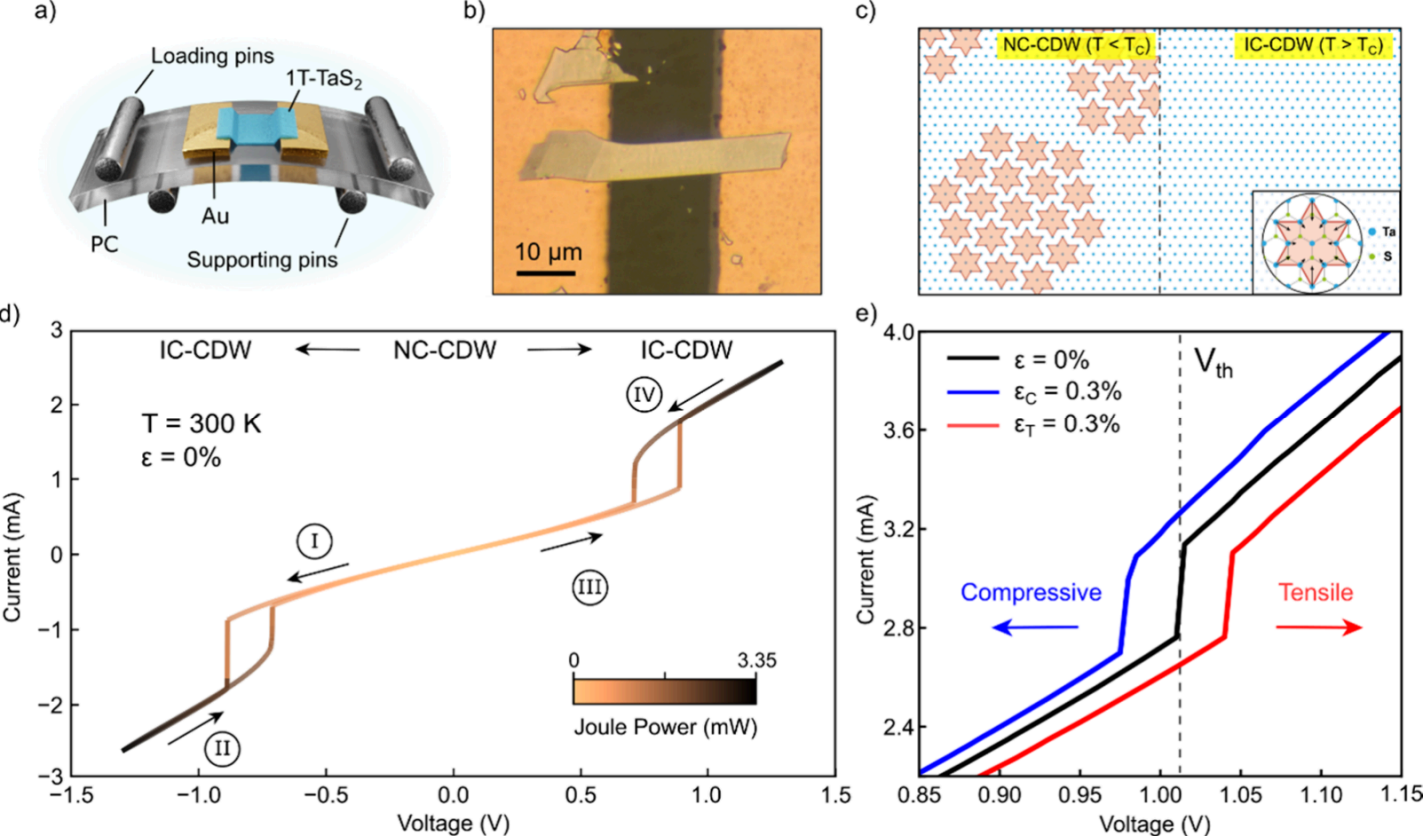
(a) Experimental concept. A two-terminal 1T-TaS_2_ device fabricated on polycarbonate (PC) is uniaxially strained using a four-point bending setup. (b) Optical image of a typical 1T-TaS_2_ flake in the two-terminal configuration. (c) Schematic of the charge density wave (CDW) phases of 1T-TaS_2_ near RT. In the nearly commensurate (NC) phase, Star-of-David supercells (see the inset) form hexagonal domains. Above T_C_, the CDW becomes incommensurate (IC). (d) Current–voltage (*I–V*) characteristic of Device 1 under zero applied strain. The NC-to-IC phase transition, driven by Joule heating, occurs at threshold voltage *V*_th_, appearing as an abrupt current increase in curve I, with hysteresis observed upon reducing the bias in curve II (similarly for positive voltages in curves III and IV). (e) Strain tuning of the NC-to-IC transition in Device 2. Tensile strain increases *V*_th_, while compressive strain decreases *V*_th_. For the sake of visual clarity, only the forward sweeps are shown.

At room temperature (*T* < T_C_), the 1T-TaS_2_ exhibits a nearly commensurate charge density wave (NC-CDW), characterized by hexagonal domains of CDW supercells arising from a $\sqrt{13}$ × $\sqrt{13}$ Star-of-David distortion of the Ta atoms (Figure 1c). In the NC-CDW state, electronic conduction largely takes place along incommensurate regions between the commensurate CDW domains. As the temperature increases above T_C_ ≈ 350 K, the CDW order becomes incommensurate with the underlying lattice, and the material enters a metallic incommensurate CDW (IC-CDW) phase. The proximity of this phase transition to RT makes 1T-TaS_2_ an ideal platform for controlling CDW order under ambient conditions.

Interestingly, this NC-to-IC phase transition can be driven by Joule heating, as shown in Figure 1d. The current–voltage (*I–V*) characteristics of the flake exhibit a sharp, abrupt increase in current around ± 0.85 V during the forward voltage sweeps (I and III). This current jump signals the transition to the IC-CDW phase, as the dissipated Joule power raises the flake temperature above T_C_. When the voltage is swept back down (II and IV), the system returns to the NC-CDW phase at around ± 0.72 V. The hysteresis observed between forward and backward sweeps reflects the thermal nature of the transition. This behavior is consistent with previous reports on devices fabricated on rigid substrates.^35,36,50,56,57^ All measurements presented here are performed at ambient conditions, with the only source of heating being self-heating, i.e. the electrical power dissipation within the device.

We now explore how uniaxial strain modifies this heating-induced phase transition. To do so, we perform *I–V* measurements while systematically applying either tensile or compressive strain to the substrate with a four-point bending setup (see Methods). Figure 1e shows representative *I–V* curves of the same device (Device 2) for three different strain conditions: zero strain, tensile strain (*ε*_*T*_ = 0.3%), and compressive strain (*ε*_*C*_ = 0.3%). While compressive strain *ε*_*C*_ is usually defined as negative, in this work we will refer to the absolute value of each type of strain, i.e. we define both *ε*_*T*_ and *ε*_*C*_ as positive.

In the three cases, an abrupt jump in the current, marking the NC-to-IC phase transition, is clearly visible. Notably, the threshold voltage at which the phase transition occurs, denoted *V*_th_, is shifted by the applied strain. In the absence of strain *V*_th_ ≈ 1.01 V, while tensile strain increases *V*_th_, delaying the onset of the phase transition. Conversely, compressive strain reduces *V*_th_, promoting an earlier transition.

To further investigate this tunability, we systematically study how *V*_th_ evolves as a function of applied strain. Figure 2 shows *I–V* characteristics obtained while progressively increasing the applied tensile and compressive strain. We show here two separate devices for tensile (Device 3) and compressive strain (Device 4). For tensile strain (Figures 2a–b), we observe a clear, monotonic increase in *V*_th_ as the flake is stretched. A linear fit to the data up to 0.6% strain yields a gauge factor (for the threshold voltage) of Δ*V*_th_/Δε = 55 mV/%. This behavior is reversible and stable over multiple strain cycles (Figure 2c) that comprise several hours of measurement time, highlighting the robustness of the phenomenon (see Figure S8 for longer cyclic measurements).

**Figure 2:**
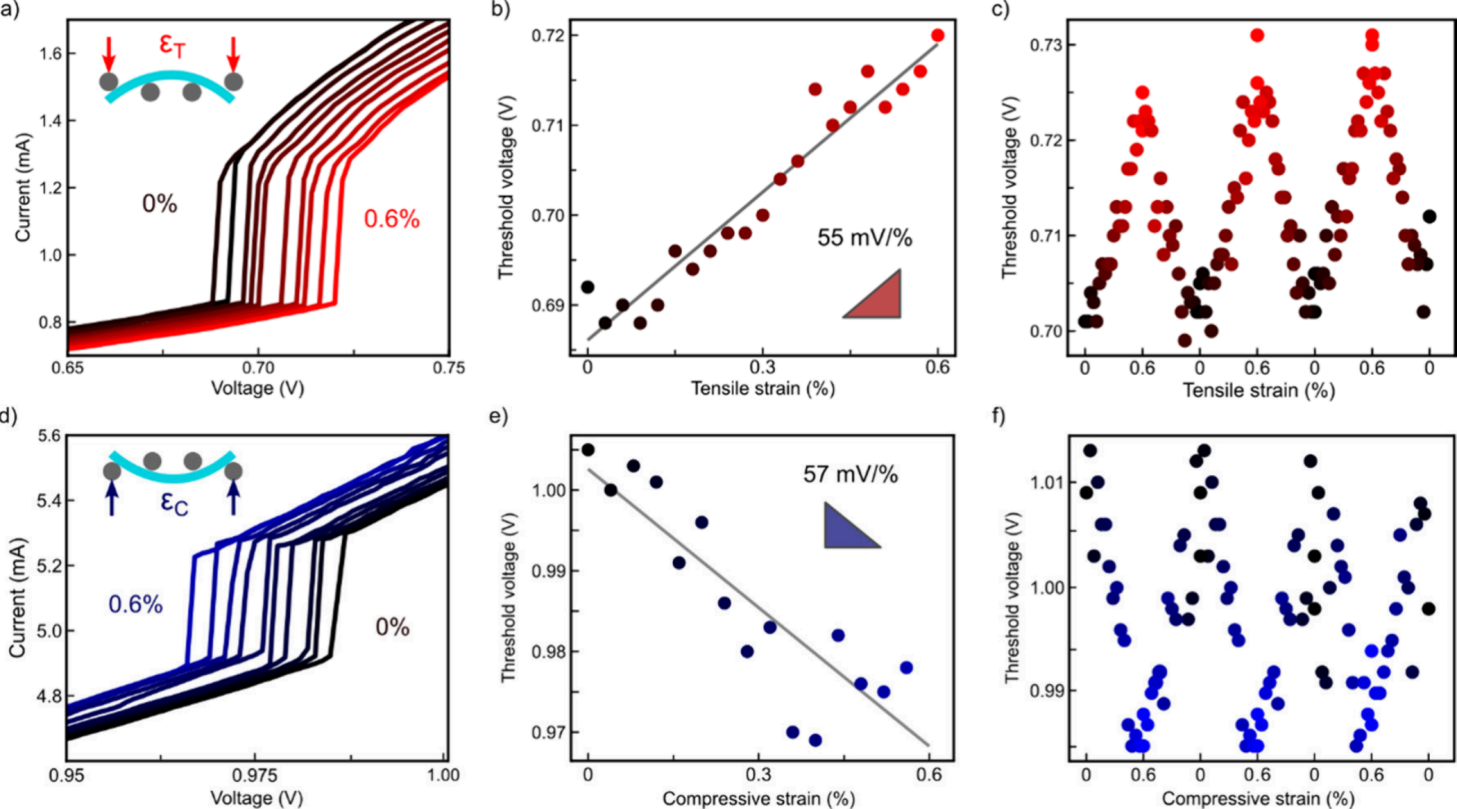
(a) *I–V* characteristics under increasing tensile strain for Device 3. *V*_th_ increases monotonically with tensile strain. (b) Linear fit between *V*_th_ and *ε*_*T*_ to extract the threshold voltage gauge factor. (c) Cyclic measurement demonstrating stable and reversible control of *V*_th_ under tensile strain. (d) *I–V* characteristics under increasing compressive strain for Device 4. *V*_th_ decreases monotonically with an increase in compressive strain. (e) Linear fit between *V*_th_ and *ε*_*C*_ to extract the threshold voltage gauge factor. *ε*_*C*_ is defined as negative, keeping the gauge factor positive. (f) Cyclic measurement of *V*_th_ under compressive strain. In panels e and f, the absolute values of *V*_th_ are extracted from the negative branch of the *I–V* curves, in contrast to panel d.

Applying compressive strain produces the opposite effect (Figure 2d), with *V*_th_ decreasing monotonically as the flake is compressed. The magnitude of the strain sensitivity (Figure 2e) under compression is similar to the tensile case, with a threshold-voltage gauge factor of Δ*V*_th_/Δε = 57 mV/%. Within the definition used here, this figure of merit remains positive, indicating that *V*_th_ decreases as the magnitude of compressive strain increases. As for tensile strain, the device shows good reproducibility over multiple strain cycles (Figure 2f). We note that in Figures 2e-f, we depict the absolute values of *V*_th_ extracted from the negative branch of the *I–V* characteristic (I < 0), while panel (d) depicts the positive branch.

These results demonstrate that uniaxial strain provides a robust and continuous method to control the NC-to-IC CDW phase transition at RT. We observe this trend consistently across all fabricated devices (additional data in Supporting Information), where the crystallographic axes of the flakes are randomly aligned with respect to the strain direction. This indicates an in-plane isotropy of the strain modulation of 1T-TaS_2_, as previously reported.^29^

In addition, we explored the effect of biaxial tensile strain on the NC-to-IC phase transition. We fabricated samples in rigid (Si/SiO_2_) and flexible (PC) substrates and compared the *I–V* characteristics as the sample temperature is increased above RT (see Supporting Information). The thermal expansion of the PC substrate (larger than that of Si/SiO_2_) exerts biaxial tensile strain on the device.^58^ The observed trend in *V*_th_ agrees with that of uniaxial tensile strain, i.e., *V*_th_ shifts to larger values as the sample is (biaxially) strained.

Beyond shifting the threshold voltage, uniaxial strain also modifies the flake resistance. We examine the piezoresistance of the NC-CDW phase as it could be linked to the strain tunability of the NC–IC transition. Figures 3a and 3b show the strain-dependent resistance of the NC phase under tensile and compressive strain, respectively: tensile strain increases the resistance approximately linearly, while compression reduces it, yielding a positive gauge factor.

**Figure 3:**
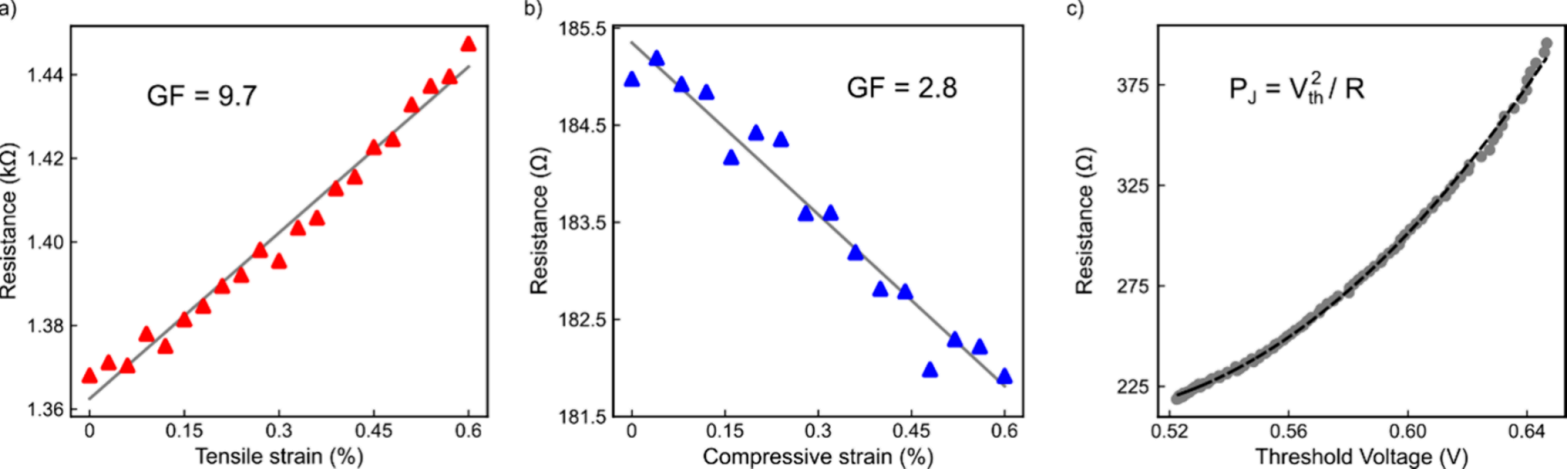
(a) Resistance of Device 3 in the NC-CDW state under increasing tensile strain. The resistance increases linearly, resulting in a positive gauge factor (GF). (b) Resistance of Device 4 under increasing compressive strain. The resistance decreases, but the gauge factor remains positive, as compressive strain is defined as negative. (c) Quadratic relation between the resistance and threshold voltage for Device 5 under uniaxial tensile strain (*R* ∝ *V*_th_^2^), confirming that strain-dependent Joule heating governs the NC-to-IC phase transition.

This response likely originates from a geometric effect that typically dominates piezoresistivity in metals. In the NC phase of 1T-TaS_2_, transport proceeds along a percolative network between commensurate domains.^42^ Stretching the flake increases the effective path length and reduces its width (via the Poisson effect), producing the observed positive gauge factor. Other contributions, such as strain-induced changes to the density of states or phonon spectrum, are not expected to significantly affect room-temperature resistance. The piezoresistance of the IC-CDW phase (see Supporting Information) is consistent in sign and magnitude with the NC-CDW response.

We quantify the strain sensitivity of the resistance using the piezoresistive gauge factor, defined as GF = Δ*R*/*R*_0_ *ε*), where Δ*R* is the strain-induced change in resistance, *R*_0_ is the resistance under zero strain, and ε is the strain magnitude. We find GF = 9.7 for tensile strain and GF = 2.8 for compression, in line with typical geometric gauge factors of GF ≈ 1–10 for metallic systems.^59^ Such piezoresistive effect enables 1T-TaS_2_ devices to function as bipolar piezoresistive strain gauges, which can detect both tensile and compressive strain continuously. Notably, the devices exhibit the same sign and comparable magnitude of piezoresistance both below and above the NC-to-IC phase transition, enabling operation in either phase. Furthermore, measurements on additional devices reveal larger values for the piezoresistive gauge factor, in the order of GF ≈ 100 (see Supporting Information). Compared to other single-flake 2D piezoresistive strain gauges,^60–62^ our devices deliver comparable gauge factors, operate at large strain levels and feature far simpler device architectures.

The observed strain dependence of both resistance and *V*_th_ suggests an intuitive origin for the strain-tunability of the phase transition. The Joule power dissipated in the device, given by *P*_J_ = *V* ^2^/*R*, depends directly on the flake resistance. Thus, as the applied tensile strain increases *R*, a higher voltage is required to reach the critical power needed to drive the transition to the IC-CDW state. Conversely, compressive strain lowers the resistance, reducing the required threshold voltage. Previous studies have established that the NC–IC transition is primarily driven by Joule heating in the channel,^35,56^ supporting our hypothesis on the origin for this strain-tunable phase transition.

Assuming that the critical Joule power (i.e., the critical temperature T_C_) remains constant with strain, this relationship implies R­(ε) ∝ *V*_th_^2^(ε). To test this hypothesis, we acquire high-resolution *I–V* data under incremental strain steps of 0.006% up to 0.6% (see Supporting Information) in an additional sample (Device 5). For each strain condition, we extract the corresponding *V*_th_ and the resistance of the flake in the NC-CDW phase. Plotting these values (Figure 3c) reveals a clear quadratic relationship between R­(ε) and *V*_th_(ε), confirming that the strain-tunability of the phase transition originates from the piezoresistive modulation of the device resistance.

Finally, we leverage the strain-tunable phase transition between CDW phases to demonstrate an alternative mechanism for detection of uniaxial strain at room temperature. We construct threshold-like strain detectors that exploit the destruction (nucleation) of charge order to sense compressive (tensile) uniaxial strain. Exploiting the bistability of the *I–V* characteristics as an amplification mechanism, similar to snap-through sensors,^63^ these detectors exhibit exceptional sensitivity and an electrically tunable detection window.

The detection principle for compressive strain is illustrated in Figure 4a. The device is first initialized in the NC-CDW state under a constant detection bias *V*_det_, selected close to the strain-dependent threshold voltage *V*_th_. As discussed earlier, compressive strain lowers *V*_th_, eventually bringing it below the fixed detection bias. When this condition is met, the dissipated power exceeds the critical value required to drive the NC-to-IC phase transition, resulting in a sharp, measurable increase in current. This threshold-like response is shown in Figure 4b: as compressive strain is gradually increased at constant *V*_det_, the device undergoes an abrupt switching event when *V*_det_ ≥ *V*_th_, producing a clear electrical signal. Notably, the strain level at which this transition occurs can be continuously tuned by adjusting *V*_det_, defining a programmable detection window for compressive strain (dashed lines in Figure 4a), which in this case extends from 0 to 0.3%.

**Figure 4:**
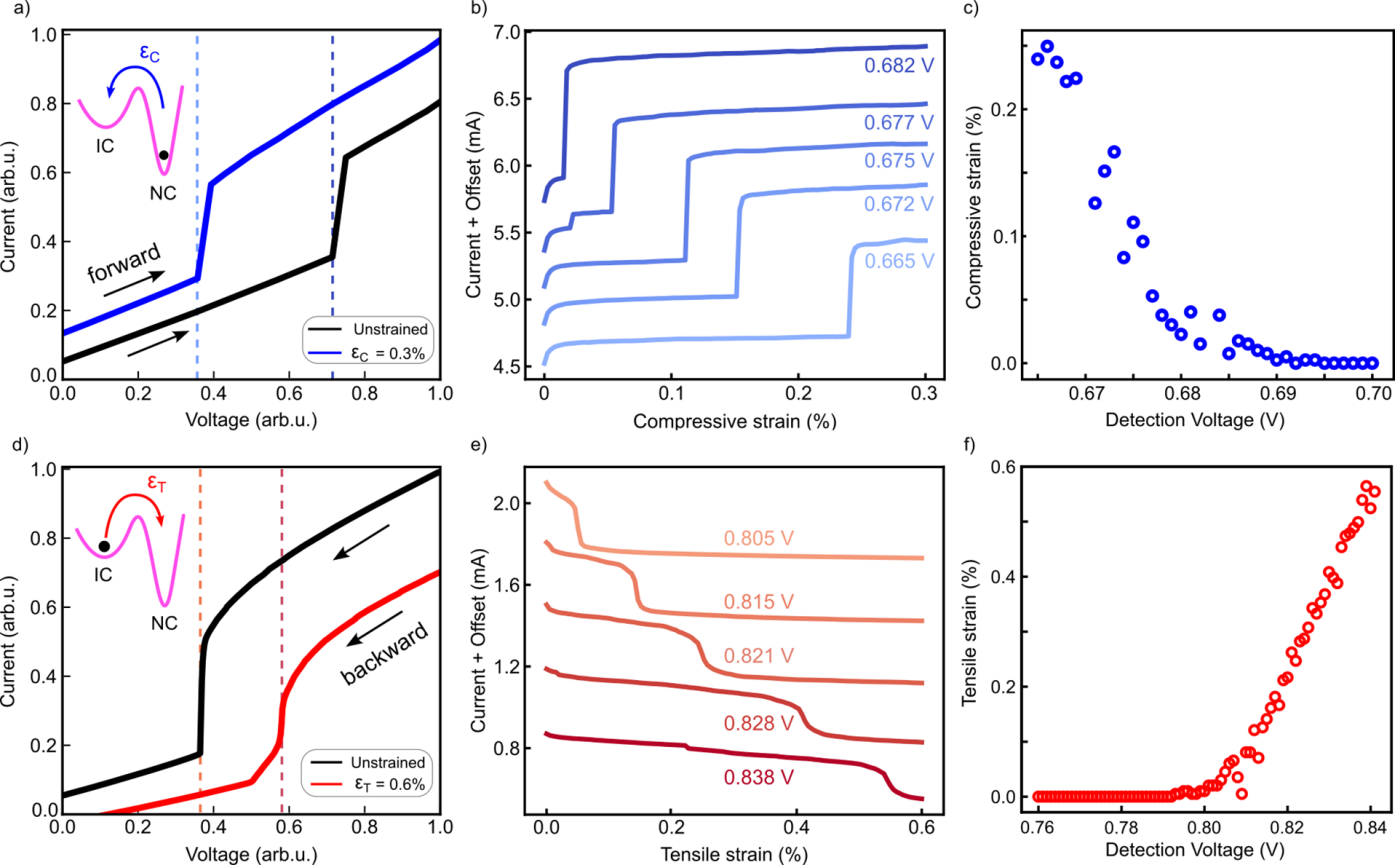
(a) Operating principle of the CDW-enhanced compressive strain detector. The flake starts in the NC state, biased at a detection voltage *V*_det_ near the phase transition (within the range indicated by the dashed vertical lines). Compressive strain reduces *V*_th_, triggering a sharp current increase as the flake switches to the IC state (see the inset). (b) Abrupt switching of the device upon incremental application of compressive strain, for different detection voltages. (c) Programmable detection window for compressive strain, tuned via *V*_det_. (d) Operating principle for the CDW-enhanced tensile strain detector. The flake is prebiased in the IC phase and held within the threshold voltage window. Tensile strain increases *V*_th_, prompting the return to the NC state. (e) Abrupt switching of the device upon incremental application of tensile strain, for different values of *V*_det_. (f) Programmable detection window for tensile strain detection.

We quantify the tunability of the detector by plotting the detectable strain as a function of the detection voltage *V*_det_ (Figure 4c). A linear fit yields a tunability of Δ*ε*_*C*_/Δ*V*_det_ = 0.0128%/mV, allowing precise control over the strain detection threshold. The current jump associated with the phase transition is sizable (Δ*I* ≈ 0.75 mA) and remains largely independent of the specific threshold conditions. Furthermore, the switching occurs abruptly within strain increments of Δ*ε*_*C*_ = 0.0025%, resulting in an exceptionally high strain sensitivity of Δ*I*/Δ*ε*_*C*_ ≈ 300 mA/%, enabled by the device’s intrinsic threshold response.

Detection of tensile strain is also tunable via the choice of the detection bias, as demonstrated in Figures 4e–f. Applying the same definition as before, we extract a detector tunability of Δ*ε*_*T*_/Δ*V*_det_ = 0.0264%/mV, while the corresponding strain sensitivity is Δ*I*/Δ*ε*_*T*_ ≈ 112 mA/%. We note that the IC-to-NC phase transition, which underpins tensile strain detection, is typically less abrupt than the NC-to-IC transition. The reverse transition (IC-to-NC) is governed by slow cooling and thermally activated nucleation and domain growth,^42^ resulting in a broader, less sharp switching response. Consequently, the device exhibits reduced sensitivity for tensile strain detection compared to the compressive strain configuration.

As a threshold strain sensor, our CDW-based device achieves a remarkable sensitivity of 300 mA/%, far exceeding other two-dimensional platforms.^60–66^ The NC–IC transition produces a sharp change in current, yielding clearly distinguishable signals. Moreover, the strain-detection window can be continuously tuned by adjusting the applied detection voltage, allowing real-time control over the strain range. All in all, our CDW-enhanced strain detector combines high sensitivity with straightforward integration into on-chip device architectures. The main trade-off is that after each detection event, the device remains in the switched state (IC state for compressive strain detection, NC for tensile strain detection). Therefore, the device must be reset before it can detect a new event. Negative feedback circuits,^67^ which automatically apply a reset signal after each detection event, would be required to support fast, free-running detection of strain fields.

Interpreting uniaxial strain as a change in source–drain separation (*L* ≃ 25 μm), the device functions as a high-resolution displacement sensor. Under compressive loading, it exhibits a current-displacement sensitivity of Δ*I*/Δ*L* ≈ 12 μA/pm (and 4.4 μA/pm under tension), while the displacement-detection voltage slope is Δ*L*/Δ*V*_det_ ≈ 660 pm/mV (and 320 pm/mV in tension). These parameters enable reliable detection of subnanometer motions (down to Δ*L* = 0.625 nm) within a voltage-controlled sensing window. Compared to leading resistive and piezoresistive displacement sensors, which achieve sensitivities around 1 V/μm,^68–70^ our CDW detector offers superior sensitivity (600 V/μm assuming a 50 Ω load), albeit over a limited ± 15 nm range. Nonetheless, its compact, on-chip footprint and dual operation modes for strain and displacement sensing make it a versatile platform for precision measurements in integrated 2D-material systems.

In conclusion, we have demonstrated reversible, room-temperature control of the NC-to-IC charge density wave phase transition in thin 1T-TaS_2_ flakes through the application of uniaxial strain. By systematically tuning the strain and monitoring both the transition threshold and device resistance, we experimentally confirm that the strain-dependence of the phase transition originates from the piezoresistive modulation of the flake’s resistance, which directly affects the Joule-heating conditions required to drive the transition. This mechanism enables precise and systematic tuning of the NC-to-IC phase transition via tensile and compressive strain, with tunabilities of 55 mV/% and 57 mV/%, respectively.

Leveraging the sharp, threshold-like electrical response at the phase transition, we realize a compact, highly sensitive strain and displacement detector (∼ 0.1–0.3 A/% and ∼ 10 μA/pm) with an electrically programmable detection window. Notably, one can sense subnanometer displacements at room-temperature in an on-chip architecture. Compared to conventional approaches, this platform offers a unique combination of sensitivity, tunability, and device simplicity, made possible by the intrinsic properties of CDW order in 1T-TaS_2_. Beyond strain sensing, these results establish strain-tunable CDW devices as promising candidates for threshold-driven functionalities in emerging technologies such as neuromorphic computing^71,72^ and phase-switch electronics.^73,74^ Toward practical devices, we note that polymer encapsulation^75^ presents a promising avenue to improve further the strain transmission and device stability over time.

## Use of AI Language Models

The instrumentation control software for the strain-electrical measurements was developed with AI assistance, following an autonomous-instrumentation workflow.^79^

ChatGPT (GPT-4o, OpenAI’s large-scale language-generation model) has been used to improve the English grammar and writing style of this manuscript. The authors have reviewed, edited, and revised the ChatGPT generated texts to their own liking and take ultimate responsibility for the content of this publication.

## Supplementary Material



## Notes

Details on the sample fabrication and the experimental techniques, images of flake identification and stacking, additional data sets under tensile and compressive uniaxial strain, additional data and discussion of the effects of biaxial strain, discussion of the piezoresistive origin of the transition tunability, examples of multistep transitions for flakes with inhomogeneous thickness, additional data sets on longer strain cycles, additional data of devices featuring larger gauge factors, additional data of the piezoresistive effect in the IC-CDW phase, additional data and images of the mechanical stress and fracture of the devices, additional data sets of the detection of tensile and compressive uniaxial strain, as well as a table comparing other methods for the application of strain that are compatible with transport measurements (PDF)
