# Supplementary material for: Strain Engineering of Correlated Charge-Ordered Phases in 1T-TaS2
Source: Nano Lett. 2025 Oct 30;25(46):16379–86. doi: 10.1021/acs.nanolett.5c04101 (PMC12636080; doi:10.1021/acs.nanolett.5c04101)
Supplement: Supplementary file 1 [file nl5c04101_si_002.pdf]

Supporting Information for:

Strain Engineering of Correlated Charge-Ordered Phases in 1T-TaS_2

Rafael Luque Merino[1], Felix Carrascoso[1], Eudomar Henríquez-Guerra[2], M. Reyes Calvo[2,3], Riccardo Frisenda[4] and Andres Castellanos-Gomez[1]

[1]2D Foundry Research Group. Instituto de Ciencia de Materiales de Madrid (ICMM-CSIC), Madrid, E28049, Spain

[2]BCMaterials, Basque Center for Materials, Applications and Nanostructures, 48940 Leioa, Spain;

[3]IKERBASQUE, Basque Foundation for Science, 48009 Bilbao, Spain

[4]Dipartimento di Fisica, Università di Roma "La Sapienza", I-00185 Roma, Italy

rafael.luque@csic.es

andres.castellanos@csic.es

Table of contents

1. Sample fabrication and experimental protocols
2. Flake identification & stacking
3. Additional data for uniaxial tensile strain
4. Additional data for uniaxial compressive strain
5. Effect of biaxial strain
6. Piezoresistive origin of the strain-tuning effect
7. Multi-step transition due to non-uniform flake thickness
8. Long cycles of tensile strain on a 1T-TaS_2 device.
9. Devices with larger piezoresistive gauge factor
10. Piezoresistance in the IC-CDW phase
11. Mechanical stress and fracture of the devices
12. Additional data for detection of uniaxial tensile strain
13. Additional data for detection of uniaxial compressive strain
14. Comparison with other transport-compatible strain platforms

1. Sample fabrication and experimental protocols

Device Fabrication

The 1T-TaS_2 crystals were purchased from HQ Graphene (HQ Graphene, The Netherlands). and exfoliated using commercial scotch tape. To achieve thin crystallites suitable for device fabrication, the cleaved crystals were transferred to a polydimethylsiloxane (PDMS) carrier substrate (Gel-Film WF ×4 6.0mil, GelPak) by gently pressing the exfoliation tape against the PDMS surface and peeling it away slowly. This process ensures that a variety of flake thicknesses are available for further selection.

The identification of suitable flakes was carried out using transmission-mode optical microscopy, where semi-transparent flakes were chosen, with thicknesses around ~ 30 nm. The NC-to-IC phase transition in 1T-TaS_2 is known to be largely thickness independent[34,71]. We observe that thicker samples tend to show smaller voltage jumps at the phase transition. As the flake thickness increases, interlayer coupling becomes more important, and we hypothesize that the NC–IC switching dynamics becomes less abrupt as additional conduction channels (including out-of-plane channels) become available.

The selected flakes were then transferred from the PDMS carrier substrate to the final device substrate using an all-dry deterministic transfer method[76,77]. The transfer process was conducted under an optical zoom lens system to ensure proper alignment of the flake over the electrodes. The final substrate consists of a 250 μm thick polycarbonate sheet (Modulor, Article No.0262951) with pre-patterned source and drain electrodes. The polycarbonate substrate, chosen for its flexibility, allowed for the application of controlled strain via mechanical bending. The electrodes were fabricated by evaporating a 45 nm layer of gold onto the polycarbonate substrate, using a 5 nm titanium layer as an

adhesion promoter. Both layers were deposited using e-beam evaporation through a commercially available shadow mask (Ossila, Product Code E291) to define the electrode pattern.

Strain Application and Electrical Measurements

In a four-point bending geometry, uniaxial strain is induced in the sample by applying a force at the sides of the flexible chip (at the loading pins) while supporting it at the center (supporting pins). Depending on the sign of the applied uniaxial strain, the loading pins apply the force downwards (tensile) or upwards (compressive). The configuration for tensile uniaxial strain is pictured in Figure 1a, while the configuration for compressive strain is shown as an inset in Figure 2d. This configuration creates a bending moment, resulting in a curvature of the sample. For equidistant pins, the strain (ϵ) on the surface of the sample can be expressed as:

$$\epsilon = 27 \cdot t \cdot \Delta / 5d^2,$$

where t is the thickness of the sample, Δ is the vertical deflection at the midpoint and d is the distance between any two consecutive pins.

Uniaxial strain was applied using a motorized bending setup capable of very precise displacements of the pivotal points of the bending apparatus. The strain levels ranged from 0.0% to 1%, and were previously calibrated by following the protocol described in previous work[55,78]. In the four-point bending used to apply uniaxial tension and compression; reversing the strain sign requires remounting the sample from the tensile to the compressive configuration. During this process, inadvertent strain can induce flake slippage or fracture (see Supp. Info.). To avoid this artifact, we focus on separate devices for tensile and compressive measurements, in order to study their pristine properties. We note that, among other methods to apply strain to transport devices, four-point bending

represents a simple, yet powerful method that enables us to dynamically apply strain in a repeatable manner (see Figure S8). In Table 1 of the Supp. Info., we provide a brief comparison with other established strain methods that are compatible with electrical transport.

Electrical measurements were carried out using a Keithley 2450 source meter unit to perform current-voltage (*IV*) sweeps while progressively increasing strain. The CDW transition was induced via Joule heating, with the threshold voltage corresponding to the point at which the current exhibited a sudden increase, signaling the phase transition.

Strain-Induced Switching

To investigate strain-induced switching, we fixed the voltage within the transition range, based on the *IV* characteristics, and monitored the source-drain current as a function of strain. This allowed us to observe abrupt current changes associated with strain-induced modifications to the CDW phase. In the case of tensile strain, as the transition moves to higher voltages with increasing strain, the flake is pre-biased to the high-temperature IC-CDW phase and we exploit the hysteretic nature of the phase transition. Thus, we detect switching from the IC-CDW phase to the NC-CDW upon application of tensile strain.

Biaxial strain measurements

Biaxial strain measurements were performed in an Attodry800 cryostat under fixed pressure of 10^{-3} mbar. *IV* characteristics were measured using a National Instruments NI USB-6343 data acquisition board to apply the source-drain voltage and record the current flowing through the sample, upon amplification by a Femto DLPCA-200 current amplifier. The *IV* sweeps were performed at a rate of approximately 21 mV/s for both samples, measuring one after the other once the target temperature was reached. The

fabrication of samples on rigid Si/SiO_2 substrates followed the same procedure detailed above.

2. Flake identification & stacking

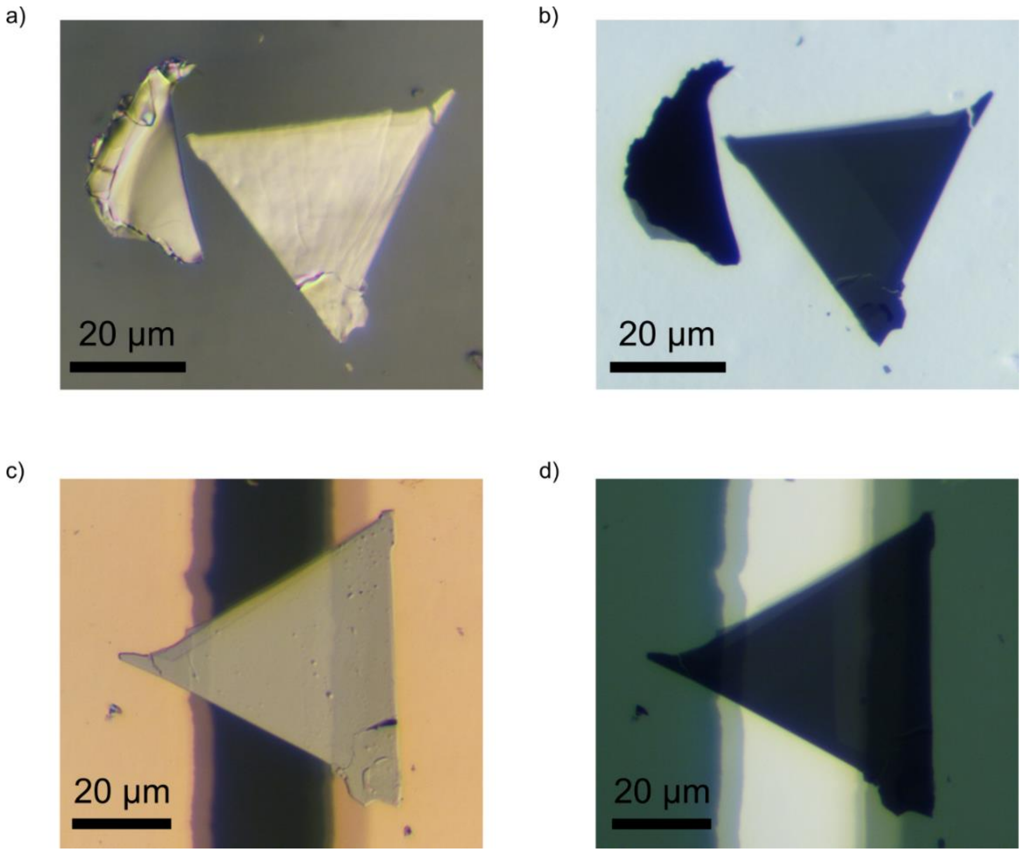

Figure S1. a) Reflection optical image of the as-exfoliated 1T-TaS_2 flake in PDMS Gel-Film WF b) Transmission mode optical image of the same flake. c) Reflection optical image of the 1T-TaS_2 flake placed between metallic electrodes on a PC substrate. d) Transmission mode optical image of the flake across the metallic leads.

3. Additional data for uniaxial tensile strain

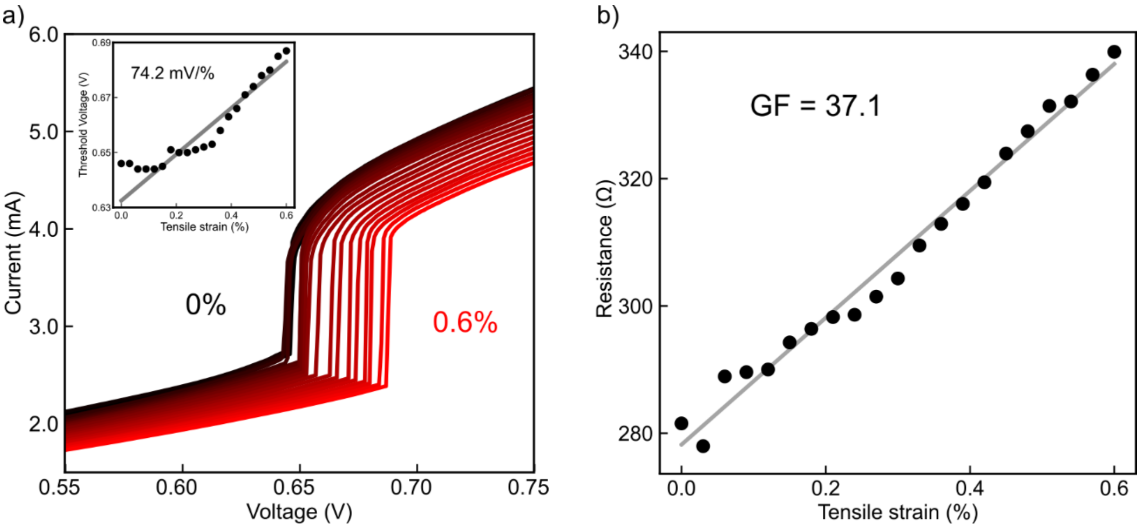

Figure S2. a) Current-voltage characteristics of a 1T-TaS_2 device for increasing tensile strain. As discussed in the Main Text, the threshold voltage for the NC-IC phase transition shifts to higher voltages as the flake is stretched. Inset shows the sensitivity of the threshold voltage to the applied strain. b) Piezoresistance of the 1T-TaS_2 device under applied tensile strain. We observe a positive piezoresistance, characterized by a large value of the piezoresistive gauge factor.

4. Additional data for uniaxial compressive strain

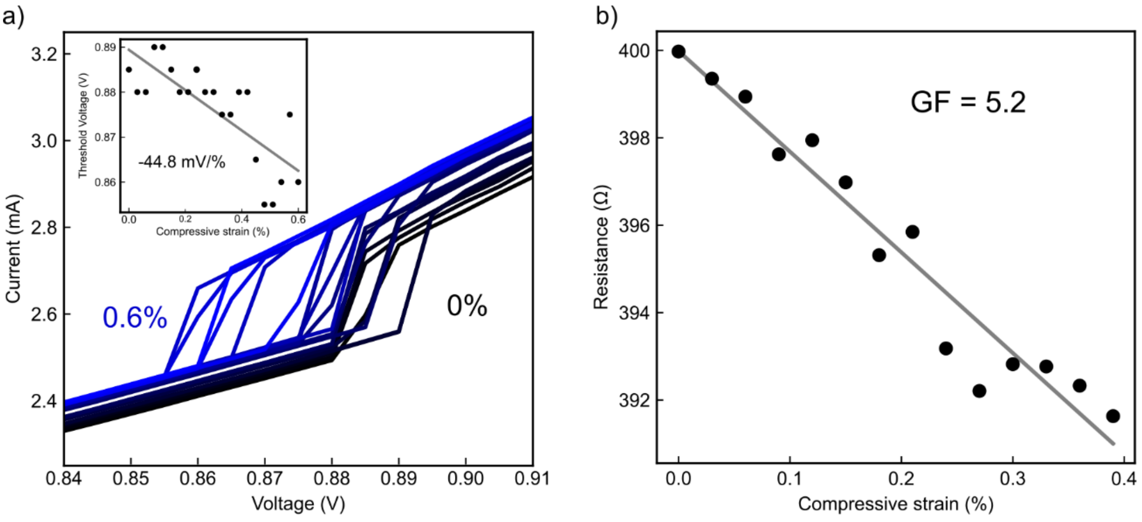

Figure S3. a) Current-voltage characteristics of a 1T-TaS_2 device for increasing compressive strain. As discussed in the Main Text, the threshold voltage for the NC-IC phase transition shifts to lower voltages as the flake is compressed. Inset shows the sensitivity of the threshold voltage to the applied strain. b) Piezoresistance of the 1T-TaS_2 device under applied compressive strain. We observe a negative piezoresistance, characterized by a moderate value of the piezoresistive gauge factor.

5. Effect of biaxial strain

We briefly explore the effect of biaxial strain on the NC-to-IC phase transition. To this end, we compare *IV* characteristics on two-terminal devices fabricated on PC and Si/SiO_2. Upon sample heating, between 300 K and 320 K, the PC substrate will undergo thermal expansion of 0.06 %, which effectively applies biaxial tensile strain to the 1T-TaS_2 flake. We estimate the magnitude of the biaxial tensile strain transferred to the flake to be ≈ 0.05%, based on an independent calibration (to be published). In contrast, the sample fabricated on Si/SiO_2 will experience negligible thermal expansion, and thus it will be considered the "unstrained" case. Figure S4 presents the numerical derivatives of the temperature-dependent *IV* characteristics for both devices, where the NC-to-IC phase transition appears as a distinct feature at the threshold voltage V_{th}.

The evolution of V_{th} with temperature is presented in Figure S5 and gauge factors are extracted as the slope $\Delta V_{th}/\Delta T$ from said data. Two competing effects determine the evolution of threshold voltage with increasing T. On the one hand, as the sample temperature is increased, less Joule heating is required to drive the flake above T_C so the threshold voltage decreases as the sample is heated up. This trivial effect explains the obtained gauge factor $\Delta V_{th}/\Delta T$ = -11.4 mV/K in the "unstrained" sample on the Si/SiO_2 substrate, obtained as the slope of data in Figure S5. For the sample on PC, the biaxial tensile strain (induced by the thermal expansion of the substrate) counteracts the aforementioned effect.

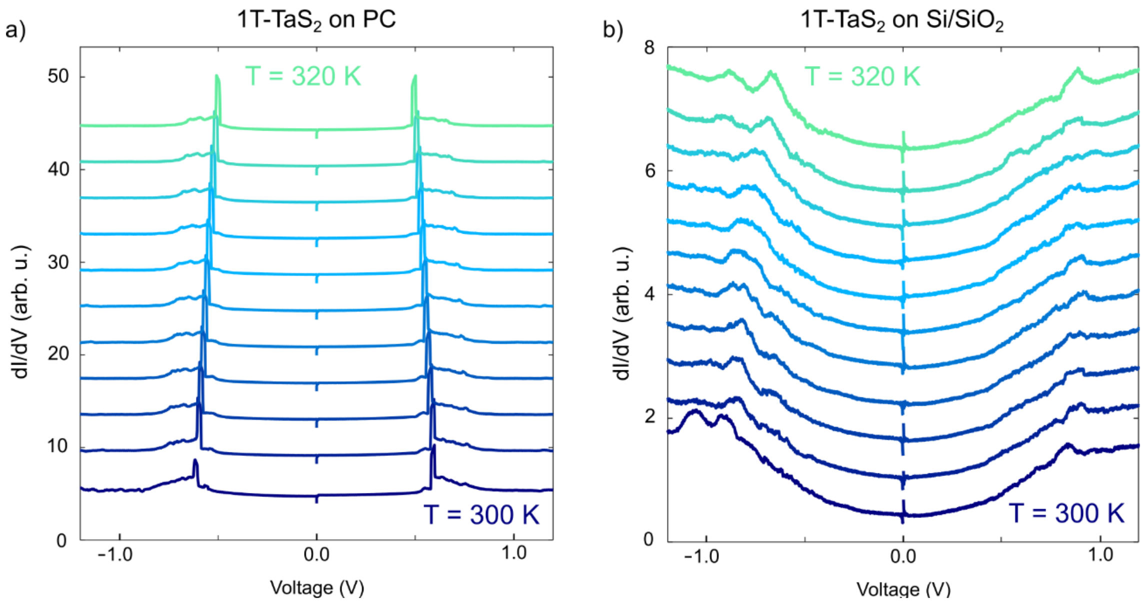

Figure S4. Numerical derivative of *IV* characteristics (dI/dV) as a function of temperature for 1T-TaS_2 device fabricated on PC. b) Numerical derivative (dI/dV) of *IV* characteristics of 1T-TaS_2 device fabricated on Si/SiO_2 as a function of temperature.

Following the results presented in this work, we posit that the piezoresistance of the flake increases with biaxial tensile strain, effectively increasing the required V_{th} to drive

the phase transition. As the effects of sample temperature and biaxial strain counteract each other in this configuration, we expect that the gauge factor in the flexible sample would be lower than for the rigid sample. Indeed, we find a lower gauge factor of $\Delta V_{th}/\Delta T$ = -5.4 mV/K for the PC device, as shown in Fig. S5.

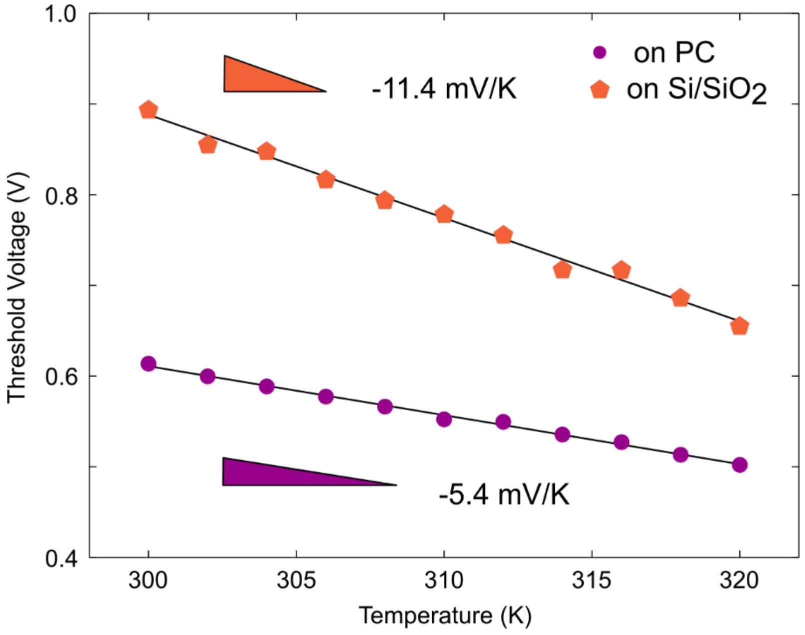

Figure S5. a) Threshold voltage vs sample temperature for 1T-TaS2 samples fabricated on SiO2/Si and PC substrates. Gauge factors are extracted as the slope of data. Overall, the increase in sample temperature reduces V_{th}. On the PC device, the biaxial tensile strain coming from thermal expansion counteracts the effect of temperature, reducing the observed gauge factor.

6. Piezoresistive origin of the strain-tuning effect

As described in the Main Text, the phase transition between the NC- and IC-CDW phases of 1T-TaS_2 can be induced by Joule heating. The heat dissipation in the current-carrying flake can increase the temperature beyond the transition temperature $T_{C,NC-IC} \approx 350$ K. Then, thermal vibrations can destroy both the hexagon-shaped clusters of $\sqrt{13} \times \sqrt{13}$ supercells that form the NC-CDW at room-temperature, as well as the individual Star-of-David supercells induced by the CDW instability.

Although this picture of heating-induced phase transitions is straightforward, other reports have focused on the application of fast electric fields, which minimize the heating effects on the system. Their findings demonstrated that strong electric fields, without significant heat dissipation, can also dissociate the nearly-commensurate order in 1T-TaS_2 via depinning and sliding of the CDW domains[36,56,78].

In this work, we apply continuous DC voltage bias across our two-terminal devices. Thus, it is expected that a build-up of heat dissipation will happen as we increase the DC bias towards the device's threshold voltage V_{th}. In the scenario of Joule heating-induced transition, one expects that the phase transition will happen at a certain threshold Joule power P_{th}^{J}, which can drive the flake above $T_{C,NC-IC} \approx 350$ K.

In our voltage-bias configuration, the Joule power can be written as $P_{th}^{J} = V_{th}^{2}/R$, where R is the device resistance. As discussed in the Main Text, we observe that applying tensile (compressive) uniaxial strain increases (decreases) both the threshold voltages V_{th} and the device resistance R. This observation is consistent with the Joule heating scenario presented above: as the device resistance increases (decreases), higher voltages need to be applied to reach the threshold Joule power that causes the NC-IC transition.

Therefore, we expect that all switching events (under zero or non-zero deformation) for a given device correspond to a pair of (V_{th}, R) values that produce the same Joule power P_{th}^{J}. To verify this hypothesis experimentally, we conducted a high-resolution measurement of a 1T-TaS_2 device (Device 5) under uniaxial tensile strain. In this measurement, shown in Fig. S6a, the tensile strain was increased from 0% to 0.6% in steps of 0.006%. The bias voltage was also finely swept in order to examine the hypothesis. Figure S6b depicts the extracted threshold voltages V_{th} and the corresponding resistance R from the NC-CDW state. Both magnitudes increase with the applied tension, as discussed throughout the Main Text.

The inset in Fig. S6b plots all the (V_{th}, R) pairs from this dataset. Here, the quadratic relation $R \propto V_{th}^{2}$ becomes apparent, confirming the origin of the strain-modulation over the phase transition. In short, the uniaxial strain modulates the flake's resistance, which in turn shifts the voltage bias needed to reach the threshold Joule power P_{th}^{J}.

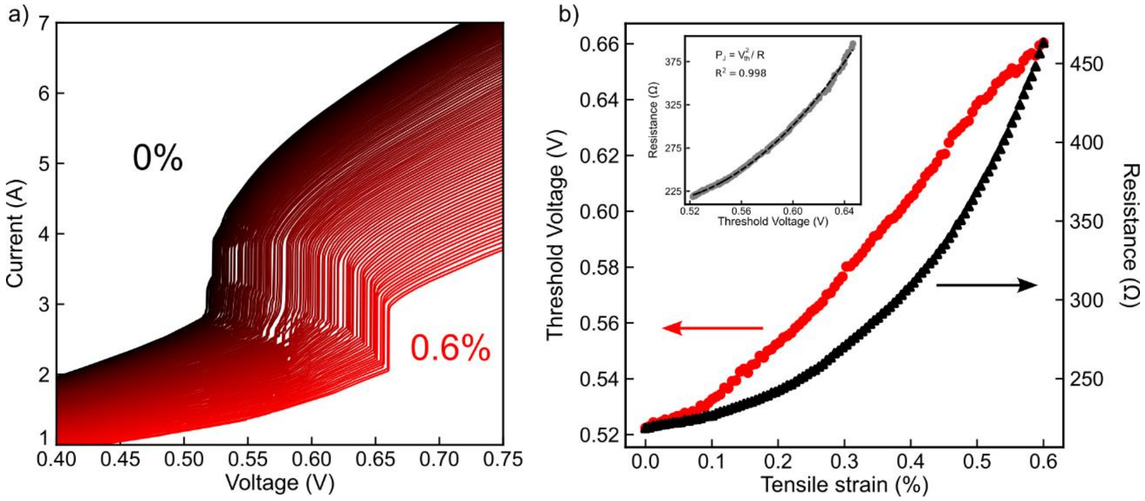

Figure S6. a) Detailed current-voltage characteristics of Device 5 under applied uniaxial tensile strain. b) Extracted threshold voltages V_{th} and resistance R in the NC state. Inset depicts the quadratic relation $R \propto V_{th}^{2}$ between all (V_{th}, R) pairs.

7. Multi-step transition due to non-uniform flake thickness

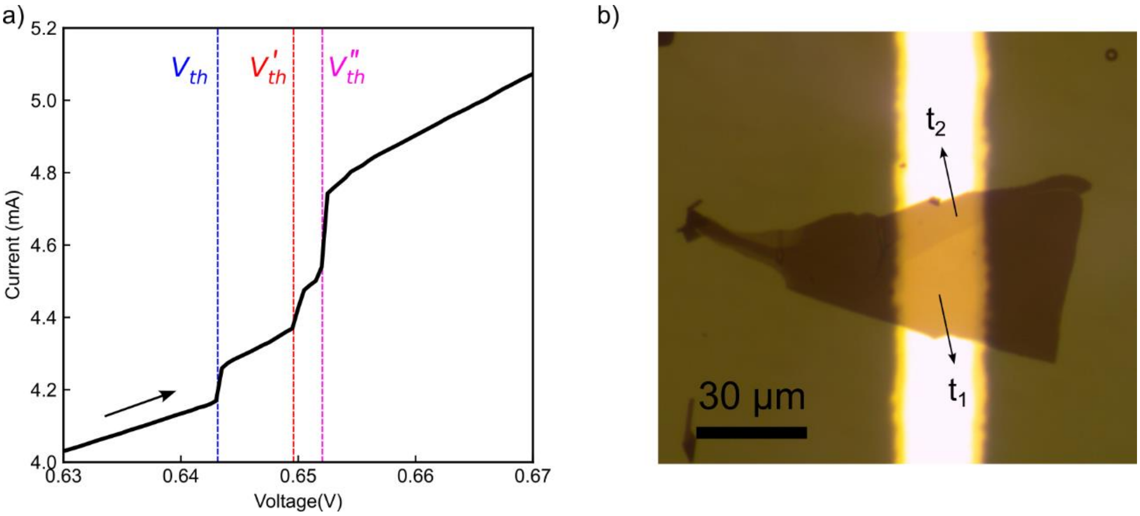

Figure S7. a) Current-voltage characteristics of a 1T-TaS_2 device featuring staircase-like jumps when approaching the NC-IC phase transition. In this device, two meta-stable intermediate states can be identified. This behavior can be observed in some samples when inducing the transition for the first time. In very few samples, this behavior can persist for multiple switching cycles. We postulate that the presence of varying flake thicknesses in the channel can lead to these multi-step transitions. b) Transmission mode optical image of a 1T-TaS_2 flake featuring multiple thicknesses (t_1, t_2) in the channel.

8. Long cycles of tensile strain on 1T-TaS_2 device.

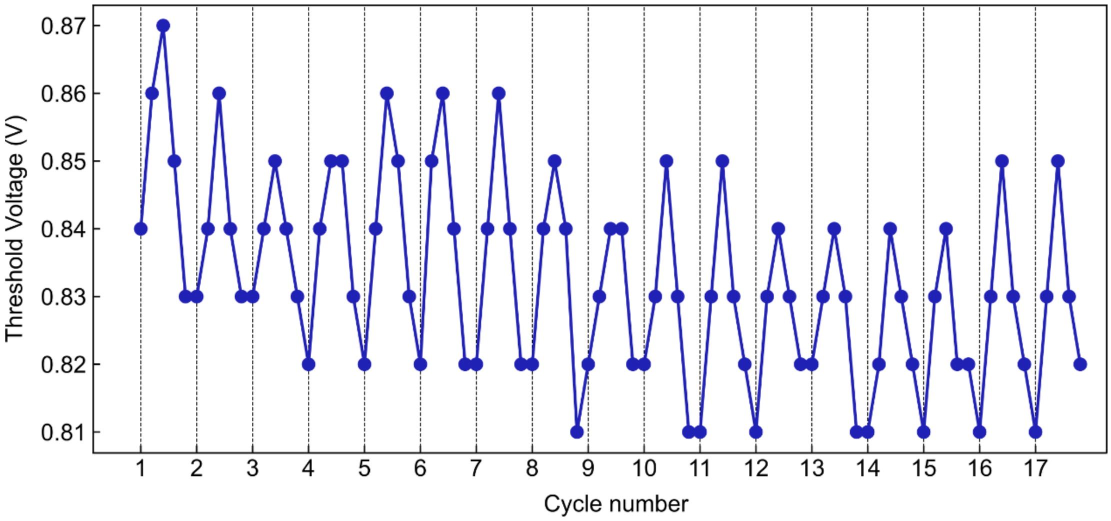

Figure S8. Threshold voltage for the switching between NC and IC phases of a 1T-TaS_2 flake, repeated over 17 cycles. V_{th} is measured at tensile strains of 0 %, 0.15 % and 0.3 %; before returning to the initial state. The vertical dashed lines mark the start of each cycle.

9. Device with larger piezoresistive gauge factor

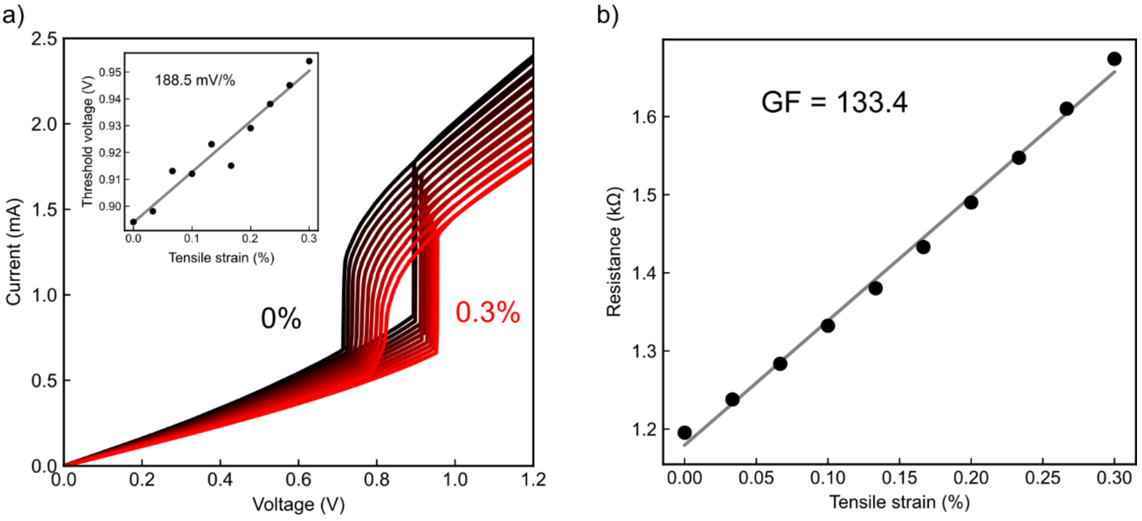

Figure S9. a) Current-voltage characteristics of a 1T-TaS_2 device for increasing tensile strain. Compared to the results shown in the Main Text, the NC-IC phase transition in this device shows very strong strain tunability. Inset shows the sensitivity of the threshold voltage to the applied strain. b) Piezoresistance of the 1T-TaS_2 device under applied tensile strain. We observe a positive piezoresistance, characterized by a very large value of the piezoresistive gauge factor.

10. Piezoresistance in the IC-CDW phase

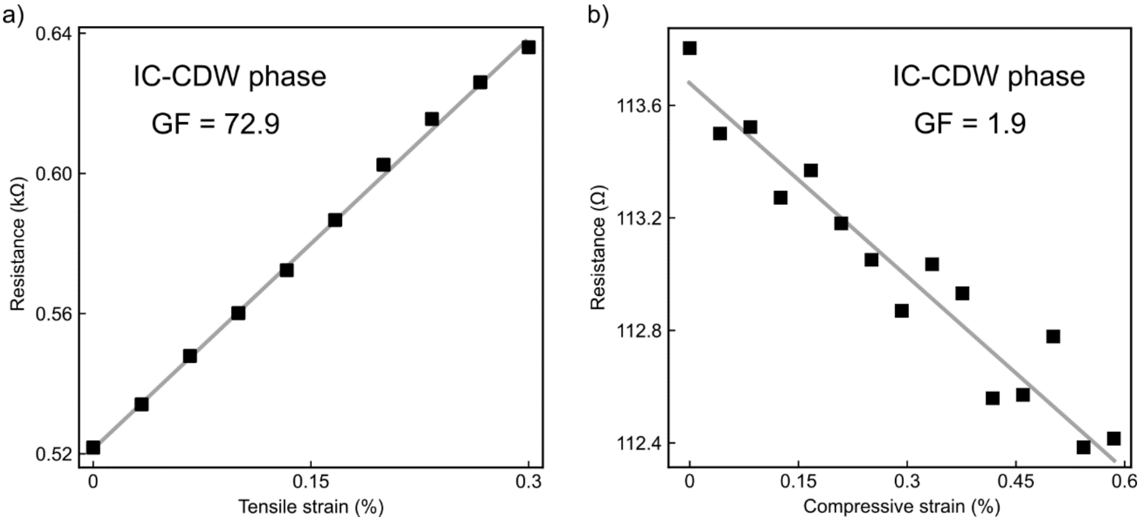

Figure S10. a) Piezoresistance of the 1T-TaS_2 flake in the IC-CDW phase under increasing tensile strain. We observe positive piezoresistance with large gauge factor, in line with the observation in the NC-CDW phase for the same flake. b) a) Piezoresistance of the 1T-TaS_2 flake in the IC-CDW phase under increasing compressive strain. We observe positive piezoresistance with moderate gauge factor, in line with the observation in the NC-CDW phase for the same flake. We note that two different devices are used for the study of tensile and compressive strain, respectively.

11. Mechanical stress and fracture of the devices

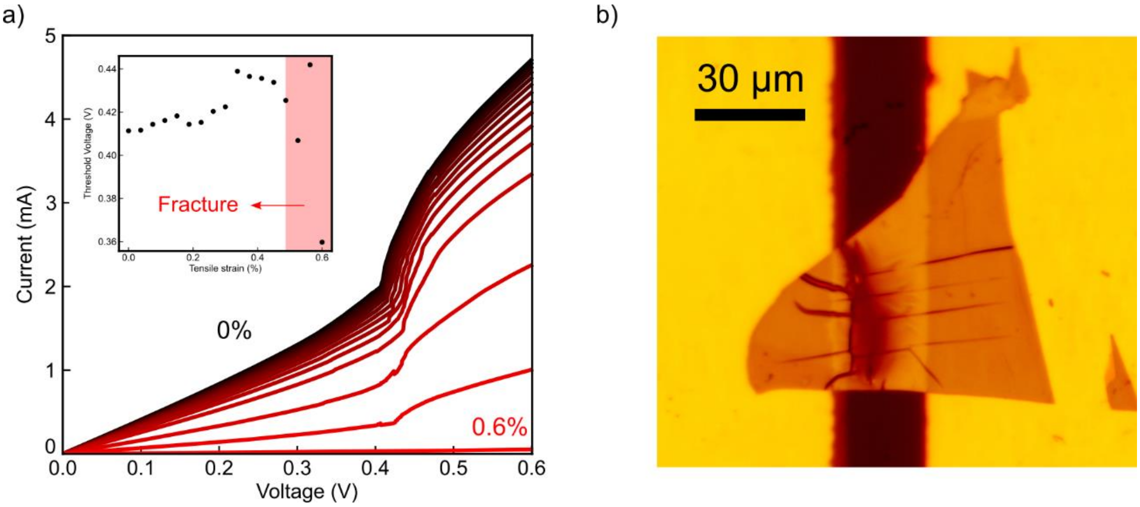

Figure S11. a) Current-voltage characteristics of a 1T-TaS_2 device for increasing tensile strain. As the applied strain is increased above 0.5%, the phase transition becomes less sharp and the device resistance increases. Upon reaching 0.6% tensile strain, the device becomes fractured and cannot sustain the source-drain current. Inset shows the evolution of the threshold voltage with applied tensile strain. The flake fracture becomes evident beyond 0.5%. b) Reflection optical image of an exemplary 1T-TaS_2 flake that has been fractured when applying uniaxial tensile strain.

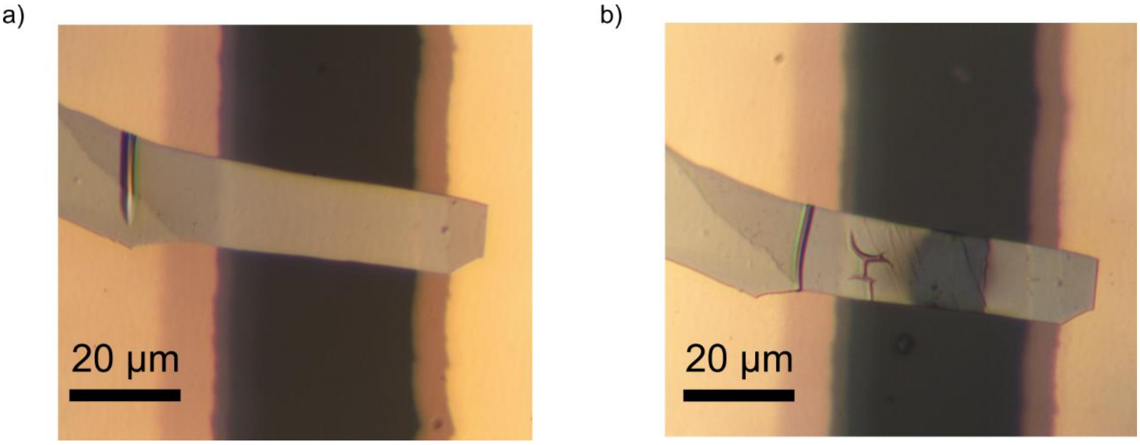

Figure S12. a) Reflection optical image of a 1T-TaS_2 flake placed between metallic electrodes on a PC substrate. b) Reflection optical image of the same 1T-TaS_2 flake as in panel (a) after its use in multiple straining cycles, spanning 12 hours of uninterrupted measurements.

12. Additional data for detection of uniaxial tensile strain

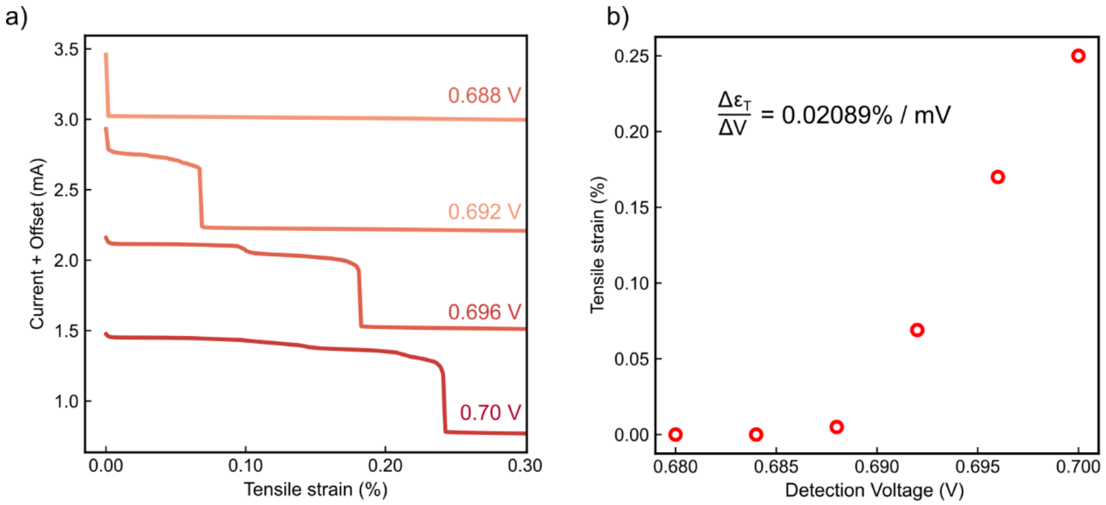

Figure S13. a) Device switching at different tensile strain levels, tuned by the choice of V_{th}. Here, the device is pre-biased to the metallic IC state and undergoes a transition to the NC state. b) Voltage-tunability of the range and sensitivity of tensile strain detection. Right vertical axis depicts the corresponding displacement sensitivity (for a 25 μm channel).

13. Additional data for detection of uniaxial compressive strain

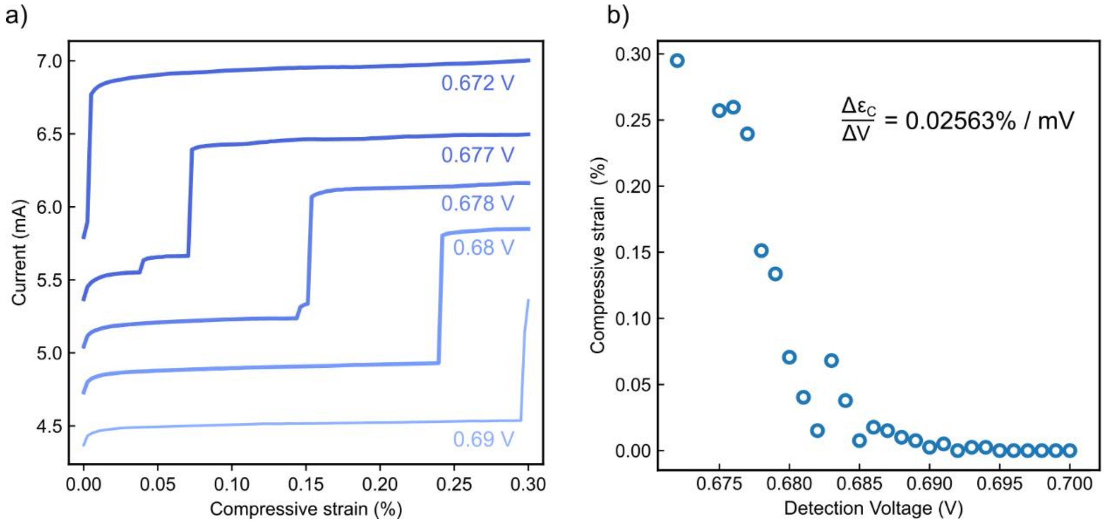

Figure S14. a) Device switching at different compressive strain levels, tuned by the choice of V_{th}. Here, the device is initially in the NC state and undergoes a heating-induced transition to the metallic IC state. b) Voltage-tunability of the range and sensitivity of compressive strain detection. Right vertical axis depicts the corresponding displacement sensitivity (for a 25 μm channel).

14. Comparison with other transport-compatible strain platforms

Method	Advantages	Limitations	Reference
**Growth-induced strain**	Scalable<br>Wafer-compatible<br>Stable over time	Resource-intensive<br>Static strain field<br>Material-specific	[7]
**Three/Four point bending**	Easy to implement<br>Material-agnostic<br>Dynamic tuning<br>Homogeneous strain	Substrate-specific<br>Hard to apply gates<br>Cryo-incompatible	[8]
**Substrate expansion/compression**	Easy to implement<br>Material-agnostic<br>Cryo-compatible<br>Homogeneous strain	Substrate-specific<br>Static strain field<br>Strain-temperature coupling<br>Only biaxial strain	[9]
**Corrugated substrates**	Easy to implement<br>Material-agnostic<br>Scalable	Substrate-specific<br>Static strain field<br>Inhomogeneous strain	[10]
**AFM tip indentation**	Material-agnostic<br>Large strain fields<br>Spatial resolution	Resource-intensive<br>Device integration<br>Only biaxial strain	[11]
**Razorbill cell**	Material-agnostic<br>Cryo-compatible<br>Good stability	Resource-intensive<br>Hard to implement<br>Mostly uniaxial	[12]
**Diamond anvil cells**	High-pressure<br>Optical access<br>Material-agnostic	Limited to hydrostatic pressure<br>Resource-intensive	[13]

Table 1. Main methods for strain-dependent transport studies.

References

[1] Yoshida, M. *et al* Controlling charge-density-wave states in nano-thick crystals of 1T-TaS2. *Sci. Rep.* **4**, 7302 (2014).

[2] Yoshida, M., Suzuki, R., Zhang, Y., Nakano, M. & Iwasa, Y. Memristive phase switching in two-dimensional 1T-TaS2 crystals. *Sci. Adv.* **1**, e1500606 (2015).

[3] Frisenda, R. *et al* Recent progress in the assembly of nanodevices and van der Waals heterostructures by deterministic placement of 2D materials. *Chem. Soc. Rev.* **47**, 53–68 (2018).

[4] Castellanos-Gomez, A. *et al* Deterministic transfer of two-dimensional materials by all-dry viscoelastic stamping. *2D Mater.* **1**, 011002 (2014).

[5] Li, H. *et al* Towards efficient strain engineering of 2D materials: A four-points bending approach for compressive strain. *Nano Res.* **17**, 5317–5325 (2024).

[6] Çakıroğlu, O., Island, J. O., Xie, Y., Frisenda, R. & Castellanos-Gomez, A. An Automated System for Strain Engineering and Straintronics of 2D Materials. *Adv. Mater. Technol.* **8**, 2201091 (2023).

[7] Ahn, G. H. *et al* Strain-engineered growth of two-dimensional materials *Nat. Commun.*. **8**, 608 (2017).

[8] Li, H. *et al* Towards efficient strain engineering of 2D materials: A four-points bending approach for compressive strain. *Nano Res.* **17**, 5317–5325 (2024).

[9] Henríquez-Guerra, E. *et al* Large biaxial compressive strain tuning of neutral and charged excitons in single-layer transition metal dichalcogenides *ACS Appl. Mater. Interf.* **15**, 57369–5737 (2023).

[10] Liu, X. *et al* Deterministic grayscale nanotopography to engineer mobilities in strained MoS2 FETs *Nat. Commun.* **15**, 6934 (2024).

[11] Huang, M. *et al* Electronic-mechanical coupling in graphene from in situ nanoindentation experiments *Nano Lett.* **11**, 1241–1246 (2011).

[12] Cenker, J. *et al* Engineering Robust Strain Transmission in van der Waals Heterostructure Devices *Newton* **1**, 100130 (2025).

[13] Bassett, W. A. Diamond anvil cell, 50th birthday *High. Press. Res.* **29**, 163–186 (2009).
